# Multi-Sensor Medical-Image Fusion Technique Based on Embedding Bilateral Filter in Least Squares and Salient Detection

**DOI:** 10.3390/s23073490

**Published:** 2023-03-27

**Authors:** Jiangwei Li, Dingan Han, Xiaopan Wang, Peng Yi, Liang Yan, Xiaosong Li

**Affiliations:** 1 Guangdong-Hong Kong-Macao Joint Laboratory for Intelligent Micro-Nano Optoelectronic Technology, School of Physics and Optoelectronic Engineering, Foshan University, Foshan 528225, China; 2 Guangdong Province Graduate Joint Training Base (Foshan), Foshan University, Foshan 528225, China; 3 Jiangsu Shuguang Photoelectric Co., Ltd., Yangzhou 225009, China

**Keywords:** medical-image fusion, embedding bilateral filter, salient detection

## Abstract

A multi-sensor medical-image fusion technique, which integrates useful information from different single-modal images of the same tissue and provides a fused image that is more comprehensive and objective than a single-source image, is becoming an increasingly important technique in clinical diagnosis and treatment planning. The salient information in medical images often visually describes the tissue. To effectively embed salient information in the fused image, a multi-sensor medical image fusion method is proposed based on an embedding bilateral filter in least squares and salient detection via a deformed smoothness constraint. First, source images are decomposed into base and detail layers using a bilateral filter in least squares. Then, the detail layers are treated as superpositions of salient regions and background information; a fusion rule for this layer based on the deformed smoothness constraint and guided filtering was designed to successfully conserve the salient structure and detail information of the source images. A base-layer fusion rule based on modified Laplace energy and local energy is proposed to preserve the energy information of these source images. The experimental results demonstrate that the proposed method outperformed nine state-of-the-art methods in both subjective and objective quality assessments on the Harvard Medical School dataset.

## 1. Introduction

The technique of image fusion integrates multiple images generated by different sensors with different descriptions of the same scene to produce an image with more compatible and accurate information [1]. The main image fusion technologies include multi-focus image fusion, medical image fusion, infrared and visible image fusion, remote sensing image fusion, etc. This technique has been widely applied in the fields of surveillance, clinical diagnostics, automation, national defense, biometrics, and remote sensing. Medical image fusion, which integrates all the useful complementary information from different medical images into a fused image, as a branch of image fusion, occupies a crucial position in research. The use of a single sensor-formed image as a basis for judgment has limitations while describing the health status of tissues (for example, using computed tomography (CT) that detects only dense structures such as bones and implants; magnetic resonance imaging (MRI) that provides soft tissue information; positron emission tomography (PET), which reflects the biological activity of cells and molecules; and single-photon emission computed tomography (SPECT), which reflects the blood flow through tissues and organs). Fused images can provide a more comprehensive, reliable, and better description of lesions, thereby making a significant contribution to biomedical research and clinical diagnosis techniques, such as surgical navigation, radiotherapy planning, and future-health prediction methods [2].

In recent years, multi-sensor medical-image fusion techniques have been developed. The four main types include: spatial domain-based (SDB), transform domain-based (TDB) [3], sparse representation-based (SRD) [4], and deep learning-based (DLB) methods [5].

The TDB method typically consists of three steps: multiscale decomposition, fusion, and multiscale reconstruction. This kind of method decomposes images into different scales, which is analogous to the process of human eyes dealing with visual information ranging from coarse to fine, enabling a better signal-to-noise ratio [6]. Zhu et al. [7] decomposed images using a non-subsampled contourlet transform (NSCT) and fused the corresponding high and low frequencies using phase congruency (PC) and local Laplacian energy (LLE), respectively. Li et al. [8] used the Laplacian redecomposition (LRD) scheme to decompose images and fused subbands based on overlapping and non-overlapping domains. In addition to selecting transform tools, fusion rules also play an integral role in TDB methods. Because fusion rules achieve image fusion by directly processing pixels or regions that tend to be considered in terms of points or regions, they cannot solve the problem of extracting edge information correctly. Moreover, TDB methods are computationally expensive and may result in inevitable losses in details and generate artifacts during decomposition and reconstruction, which could reduce the fusion performance [9,10].

SRB methods mainly work on the concept that image signals can be considered a linear combination of atoms in an overcomplete dictionary. Most SRB methods involve the following steps [4]: (a) segmenting the source image into overlapping patches, (b) sparse encoding of the patches using a dictionary to obtain their sparse coefficients, (c) combining the sparse coefficients, and (d) reconstructing the images from the sparse coefficients and a dictionary. Common sparse representation (SR) models include the traditional SR model [11], group-sparsity SR model [12], robust SR model [13], non-negative SR model [14], and joint convolutional analysis and synthesis [15]. Li et al. [11] used low-pass filtering and structured texture filtering to decompose an image and fused high-frequency layers with sparse representation to achieve image fusion and denoising. Jie et al. [16] used sparse representation and a rolling guidance filter (RGF) to fuse texture layers using cartoon–texture image decomposition. When compared to TDB methods, SRB methods allow for a more meaningful and stable representation of the source image owing to the overcomplete dictionary containing richer basis atoms. Moreover, using a fixed step size to acquire image blocks is also effective in reducing artifacts and improving robustness to misalignment. Additionally, sparse encoding under SRB methods is usually time-consuming and complex, and important information from the source images is inevitably lost [16,17].

In recent years, DLB methods have received considerable attention owing to their powerful nonlinear fitting capability. In the convolutional neural network (CNN)-based approach proposed by Zhang et al. [18], two convolutional layers were used to achieve feature extraction and the reconstruction of images. In addition to CNNs, generative adversarial networks (GANs) have also been applied to image domains. Ma et al. [19] proposed a GAN for image fusion using a generator and a discriminator to achieve maintenance of intensity and detail information in the source image. The auto-encoder-based fusion framework for feature extraction and image reconstruction uses pre-trained autoencoders. Luo et al. [20] used a multi-branch encoder with contrast constraints to learn the public and private features of an image, fused the private features using an adaptive fusion rule based on energy, and then reconstructed the image using a decoder. The end-to-end fusion process in DLB methods effectively reduces pre-processing, parameter tuning, and post-processing. However, DLB methods are time-consuming for model training and require large datasets [21,22].

SDB methods rely on detecting pixel-level activities, which reflect features such as the level of image sharpness and structural saliency. The main steps are as follows. First, the activity of a pixel or region is detected by a specific function or algorithm to obtain the activity map of the image. Then, according to a given rule (e.g., the “maximum absolute value (Abs)” rule), it generates an active decision map. Finally, the decision map is used to reconstruct the source image to obtain a fused image. In SDB methods, image processing using edge-preserving filters has become increasingly common. The base layer of the image is obtained using an edge-preserving filter to potentially capture large variations and a set of detail layers to preserve detail at progressively more refined levels. Mo et al. [23] proposed an attribute-filter-based image fusion method wherein the prominent objects in the image were first extracted using attribute- and edge-preserving filters, and then the fusion results were obtained using a weight-based Laplacian-pyramid image-fusion strategy. Overall, SDB methods are simple and fast, but pixel-level activity detection is not an easy task, and incorrect activity detection may lead to the occurrence of blocking (region) artifacts, introduce certain spectral distortions, and degrade the sharpness of the fusion results [24].

Although existing multi-sensor medical-image fusion techniques have achieved great success, certain shortcomings still exist. For example, the atoms of the dictionary in SRB methods have a limited ability to represent salient features in the image [25]. The fusion rules in TDB and SDB methods are often based on pixels or regions without consideration of edges or structures in the image [26]. In addition, most existing methods lack attention to salient information, which is often a visual reflection of tissue health status in medical images. To retain the salient information in the source images, in this paper, we propose a medical image fusion method based on least-squares using the bilateral filter (BLF-LS) and deformation smoothness constraint (DSC) [24], which can effectively retain the salient information, edge, and energy from source images.

The BLF-LS is a recently developed edge-preserving filter. It takes advantage of bilateral filtering and the least-squares (LS) model, effectively smoothing the edges within the texture region while producing results without gradient reversals and halos; it also offers the advantage of fast operation [27]. Therefore, we introduced the BLF-LS to decompose the source image. A fusion rule combining DSC and the rolling guidance filter (RGF) [25] was designed to fuse detail layers. Saliency describes what attracts the visual attention of humans in a bottom-up manner. Salient detection can maintain the integrity of important target regions and enables high-quality image fusion. The main contributions of this study are as follows:

1. A medical image fusion method based on the BLF-LS and salient detection is proposed. To the best of our knowledge, this is the first time the BLF-LS has been applied in medical-image fusion. The source images are decomposed into the detail and base layers.
2. A detail-layer fusion rule based on DSC and RGF is proposed, which fully considers the low contrast between the target and background.
3. A fusion rule based on modified Laplace energy and local energy (MLEN) was designed to maintain detailed information and energy in the base layer.
4. The proposed fusion method can be effectively extended to the IR- and VIS-image fusion problem and yield competing fusion performance.

The remainder of this paper is organized as follows. In Section 2, the background of the BLF-LS and salient detection using a DSC is briefly introduced. Section 3 explains the proposed image-fusion algorithm. The experimental results and discussion are presented in Section 4. Finally, Section 5 concludes the paper.

## 2. Related Work

### 2.1. Embedding Bilateral Filter in Least Squares

Edge-preserving filters offer many advantages, such as accurately separating image structures at different scales while maintaining the spatial consistency of these structures, reducing the blurring effects around edges, providing a good edge- and boundary-preserving performance, and smoothing background information. The BLF-LS is an edge-preserving filter achieved using global methods [27]. The smoothing result of this filter is free of gradient reversals and halos. Additionally, the BLF-LS runs faster because it utilizes the efficiency of bilateral filter (BLF) and the LS model. To facilitate the understanding of the BLF-LS, we first describe BLF. For a given image g, the output image μ through the BLF is computed as follows:

(1) $$\mu_{s}=\frac{1}{G_{\sigma_{S}}\left(\left\|s-t\right\|\right)G_{\sigma_{r}}\left(\left\|g_{s}-g_{t}\right\|\right)}\sum_{t\epsilon N\left(S\right)}G_{\sigma_{S}}\left(\left\|s-t\right\|\right)G_{\sigma_{r}}\left(\left\|g_{s}-g_{t}\right\|\right)g_{t},$$

where *s* and *t* denote different pixel points, $G_{\sigma_{S}}$ denotes the Gaussian kernel that determines the spatial support, and $G_{\sigma_{r}}$ denotes the Gaussian kernel that controls the sensitivity to the edges. The BLF has the advantage of fast image processing. However, because the edges are sharpened in the smoothed image and boosted in the reverse direction in the enhanced image, gradient inversion and halos are produced in the result.

Suppose $f_{BLF}\left(\nabla g_{*}\right)$ denotes the smoothing gradients, and ∗ denotes the axis direction of the input image g with the BLF; embedding $f_{BLF}\left(\nabla g\right)$ into the LS framework achieves efficient edge-preserving smoothing. This approach allows the BLF-LS to achieve both the edge-smoothing quality of the LS and BLF models with a proper processing efficiency, as described below. Given an input image g, the output image μ with BLF-LS is:

(2) $$\underset{\mu }{\min}\sum_{s}\left({\left(\mu_{s}-g_{s}\right)}^{2}+\lambda \sum_{*\in \left\{x,y\right\}}{\left(\nabla \mu_{*,s}-{\left(f_{BLF}\left(\nabla g_{*}\right)\right)}_{s}\right)}^{2}\right),$$

where *s* denotes the pixel position. When the value of λ is large enough, the gradient of the image *μ*, that is, $\nabla \mu_{*,s}$, will resemble $f_{BLF}\left(\nabla g_{*}\right)\left(*\in \left\{x,y\right\}\right)$, which guarantees the smooth quality of the BLF-LS. Because the LS model can be solved in the Fourier domain, the speed of the BLF-LS is guaranteed. Equation (2) can be solved as follows:

(3) $$\mu =F^{-1}\left(\frac{F\left(g\right)+\lambda \sum_{*\in \left\{x,y\right\}}\left(F\left(f_{BLF}\left(\nabla g_{*}\right)\right)\right)}{F\left(1\right)+\lambda \sum_{*\in \left\{x,y\right\}}\bar{F\left(\partial_{*}\right)}\cdot F\left(\partial_{*}\right)}\right),$$

where $F\left(\cdot \right)$ and $F^{-1}\left(\cdot \right)$ are the fast Fourier transform (FFT) and inverse fast Fourier transform (IFFT) operators, respectively; $\bar{F\left(\cdot \right)}$ denotes the complex conjugate of $F\left(\cdot \right)$; and $F\left(1\right)$ is the FFT of the delta function. Additionally, multiplication and division are both point-wise operations.

### 2.2. Salient Detection via Deformed Smoothness Constraint

The DSC [28] is a propagation model that can capture significant targets when there is low contrast between the object regions and background. It comprises three main steps. First, the image is segmented using superpixels, and the segmentation result is represented as a graph. Then, a coarse map is generated via the background seeds and a deformed smoothness-based manifold ranking model, and the objectness map is built through the object proposal. Finally, the coarse and objectness maps are used to generate a refined map.

The input image ***I*** generates a significant detection map **g** described as follows:

(4) $$\underset{g}{\min\frac{1}{2}}\left\{g^{T}\left[D^{c}-W^{c}+\mu \left(I-\frac{D^{c}}{v^{c}}\right)\right]g+\|g-M^{c}{\|}^{2}+g^{T}D^{o}g\right\},$$

where $M^{c}$ is the coarse map; $D^{c}$ and $v^{c}$ are the degree matrix and volume of $M^{c}$ respectively; $W^{c}$ is a weight matrix computed by $M^{c}$; μ is a non-negative parameter that balances the weights of the two smoothness constraints; $M^{o}$ denotes the objectness map obtained for each node using an edge box ($M^{o}={\left(m_{i}^{o}\right)}_{n}$); and $D^{o}={\left(d_{ii}^{o}\right)}_{n\times n}$ represents a diagonal matrix with $d_{ii}^{o}=diag\left(exp\left(-m_{i}^{o}\right)\right)$.

The optimal solution of Equation (4) is expressed as:

(5) $$g={\left[D^{c}-W^{c}+\mu_{2}\left(I-\frac{D^{c}}{v^{c}}\right)+D^{o}\right]}^{-1}M^{c}$$

The elements of **g** were normalized to [0, 1] and assigned to the corresponding superpixels to generate a saliency detection map.

## 3. Proposed Method

**Figure 1:**
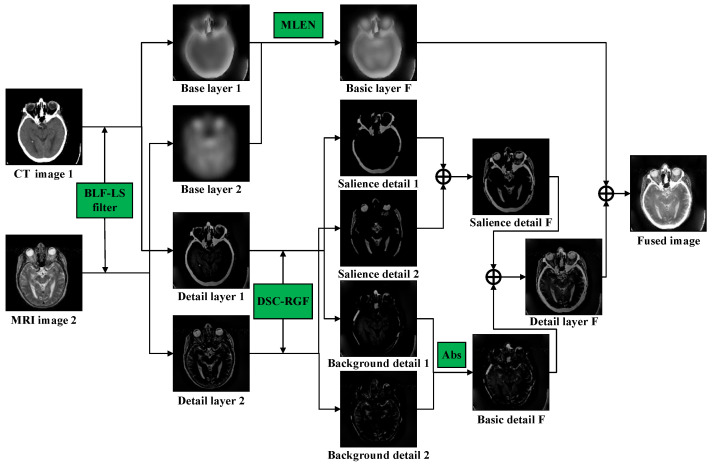
Flowchart of proposed image-fusion method.

The proposed method is illustrated in Figure 1. First, the source images are decomposed into a base layer and a detail layer via the BLF-LS. The base layer, which is obtained by decomposing the source image, is fused based on the MLEN fusion rules to retain the energy information of the source image. Moreover, the detail layers are considered a superposition of the salient regions and background information. The detail layers are decomposed into background-detail layers and salient-detail layers using a model based on the DSC and RGF. To fully retain the energy information, the background-detail layer of the fused image is obtained using the fusion rule, Abs. Regarding the salient-detail layers that contain important salient targets, the overlap between the two salient-detail layers is removed using the DSC-RGF model, and a direct summation method is used to obtain the salient-detail layer of the fused image. Finally, the fused image is obtained by reconstruction.

**Figure 2:**
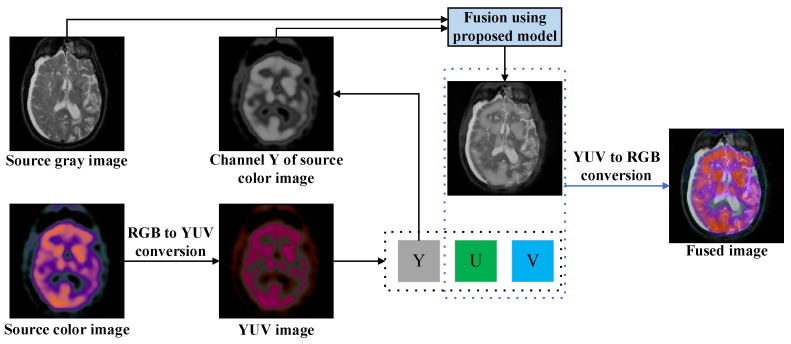
Scheme for fusing medical images with color.

Additionally, for a functional medical image fusion problem, the following conversion scheme is used: red, green, and blue (RGB)→luma, blue projection, and red projection (YUV)→RGB, as shown in Figure 2.

### 3.1. Decomposition of Base Layer and Detail Layer

BLF-LS achieves useful edge-preserving smoothing by embedding the BLF into the LS framework. First, we employed a BLF-LS to decompose the source images into a base layer and a detail layer. The details of the base layer are as follows:

(6) $$B_{n}=I_{n}*F_{BLF-LS}\;\;\;\left(n=1,\;2\right),$$

where $I_{1}$ and $I_{2}$ denote the source images, $B_{1}$ and $B_{2}$ are the base layers obtained by decomposing $I_{1}$ and $I_{2}$, respectively; and $F_{BLF-LS}$ is a *BLF−LS* used for smoothing the images described in Equation (2). After the base layer is obtained, it can be subtracted from the source image to obtain the detail layer:

(7) $$D_{n}=I_{n}-B_{n}\;\;\;(n=1,\;2),$$

where $D_{1}$ and $D_{2}$ are the detail layers obtained by decomposing $I_{1}$ and $I_{2}$, respectively. The base layer can potentially capture large variations in intensity, and the detail layer can preserve details at fine scales.

### 3.2. Decomposition of Detail Layer Based on DSC-RGF Algorithm

**Figure 3:**
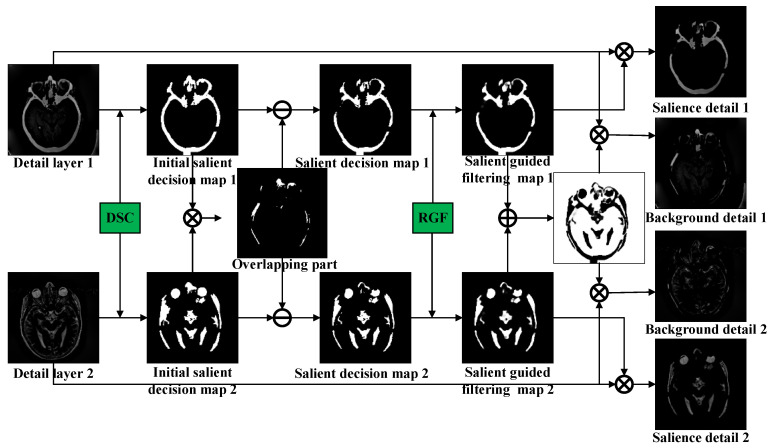
Deformed smoothness constraint–rolling guidance filter (DSC-RGF) algorithm.

In recent years, many saliency detection methods that can detect salient visual areas or objects and easily draw visual attention have been proposed. It is typically easier to detect salient targets in the detail layer obtained using a smoothing filter. To this end, we designed a method based on the DSC and RGF to fuse the detail layer, as shown in Figure 3; this method mainly consists of the following steps. First, the initial salient decision map is obtained by applying the DSC to the detail layer. Second, the overlapping part of the two initial salient decision maps produces ghosting in the fusion results and affects the visual effect; therefore, the overlap-removal procedure is performed on the initial salient decision map. Then, in view of the edge-smoothing problem of the significant target in the salient decision map, the RGF is used to process the salient decision map to obtain the salient guided filtering (SGF) map. Finally, the detail layer is decomposed into background and salient-detail layers using this map.

The details of each step are as follows. First, the DSC model is used to detect the detail layer to obtain the salient information, and threshold correction is adopted to process the salient information of the initial salient decision map.

(8) $$I_{ID}^{n}\left(x,y\right)=\left\{\begin{array}{c} 1\;\;\;\;D_{n}\left(x,y\right)*F_{DSC}\geq T\\ 0\;\;\;\;D_{n}\left(x,y\right)*F_{DSC}<T \end{array}\right.\;\;\;\;\;\;\;\;\left(n=1,\;2\right),$$

where $*F_{\mathrm{DSC}}$ denotes the salient-detection operation using the DSC model in Equation (4); *T* is the threshold value; and $I_{ISD}^{1}\left(x,y\right)$ and $I_{ISD}^{2}\left(x,y\right)$ are the initial salient decision maps obtained from $D_{1}\left(x,y\right)$ and $D_{2}\left(x,y\right)$, respectively.

Second, it is necessary to remove the overlapping part $I_{R}$, which is generated by multiplying $I_{ISD}^{1}$ and $I_{ISD}^{2}$ $\left(I_{R}=I_{ISD}^{1}\cdot I_{ISD}^{2}\right)$. Directly phasing it into the fusion result causes ghosting, which affects the visual effect.

(9) $$I_{SD}^{n}=I_{ISD}^{n}-I_{R}\;\;\;\;\;\;\;\;\left(n=1,\;2\right),$$

where $I_{SD}^{1}$ and $I_{SD}^{2}$ represent the salient decision maps obtained after removing the overlapping parts. Considering the edge-smoothing problem of the significant target in the salient decision map, we used RGF to process $I_{SD}^{n}$ to obtain the SGF map.

(10) $$I_{SGF}^{n}=I_{D}^{n}*F_{RGF}\left(T^{\prime },r,\varepsilon \right)\;\;\;\;\;\;\;\left(n=1,\;2\right),$$

where $F_{RGF}\left(\cdot \right)$ represents the RGF function; $T^{\prime }$ denotes the number of iterations; r denotes the filter size; ε denotes the degree of blur; and $I_{SGF}^{1}$ and $I_{SGF}^{2}$ denote the SGF maps used to decompose the detail-layer maps of the source image.

Finally, the salient-detail layers are obtained by multiplying the SGF maps by the detail layers, and the background-detail maps are obtained by removing the salient parts of the detail layers, as described below.

(11) $$SD_{n}\left(x,y\right)=D_{n}\left(x,y\right)\cdot I_{SGF}^{n}\left(x,y\right)\;\;\;\;\;\;\;\left(n=1,\;2\right),$$

(12) $$BD_{n}\left(x,y\right)=D_{n}\left(x,y\right)\cdot \left(1-I_{DGF}^{1}\left(x,y\right)-I_{DGF}^{2}\left(x,y\right)\right)\;\;\;\;\;\;\;\;\left(n=1,\;2\right)$$

where $SD_{1}\left(x,y\right)$ and $SD_{2}\left(x,y\right)$ denote the salient detail layers obtained from the decomposition of $D_{1}\left(x,y\right)$ and $D_{2}\left(x,y\right)$, respectively; and $BD_{1}\left(x,y\right)$ and $BD_{2}\left(x,y\right)$ denote the background detail layers obtained from the decomposition of $D_{1}\left(x,y\right)$ and $D_{2}\left(x,y\right)$, respectively.

### 3.3. Fusion of Base Layer Based on MLEN

The pair of base layers obtained under the BLF-LS decomposition contain abundant energy information and little information on the detail in the source image. Therefore, we considered using local energy (LEN) to extract the energy information and sum-modified Laplacian (SML) energy to extract the detail-related information from the base layers and finally add these two types of information to obtain the fused base layer. SML is defined as follows [29]:

(13) $$SML_{n}\left(x,y\right)=\sum_{m=-M}^{M}\sum_{n=-N}^{N}ML_{n}{\left(x+m,y+n\right)}^{2}\;\;\;\;\;\;\;\;\left(n=1,\;2\right),$$

where *M* × *N* denotes the window size centered at (*x*,*y*), and $ML_{n}\left(x,y\right)$ denotes the modified Laplacian (ML) at point (*x*,*y*); $ML_{n}\left(x,y\right)$ is defined as follows:

(14) $$ML_{n}\left(x,y\right)=\left|2B_{n}\left(x,y\right)-B_{n}\left(x-1,y\right)-B_{n}\left(x+1,y\right)\right|+\left|2B_{n}\left(x,y\right)-B_{n}\left(x,y-1\right)-B_{n}\left(x,y+1\right)\right|\;\;\left(n=1,\;2\right),\;\;\;\;\;\;\;\;\;\;\;\;\;\;\;\;\;\;\;\;\;\;\;\;\;\;\;\;\;\;\;\;\;$$

where $B_{1}$ and $B_{2}$ are the base layers decomposed from $I_{1}$ and $I_{2}$, respectively. *LEN* is defined as follows:

(15) $$LEN_{n}\left(x,y\right)=\sum_{m=-M}^{M}\sum_{n=-N}^{N}B_{n}{\left(x+m,y+n\right)}^{2}\;\;\;\;\;\;\;\;\left(n=1,\;2\right),$$

where *M* × *N* denotes the window size centered at $\left(x,y\right)$, and the fusion of the base layer can be briefly described as follows:

(16) $$B_{F}\left(x,y\right)=\left\{\begin{array}{ll} B_{1}\left(x,y\right),\; & SML_{1}\left(x,y\right)\geq SML_{2}\left(x,y\right)\;\;or\;\;LEN_{1}\left(x,y\right)\geq LEN_{2}\left(x,y\right)\\ B_{2}\left(x,y\right),\; & else \end{array}\right.$$

where $B_{F}\left(x,y\right)$ denotes the base layer of the fused image.

### 3.4. Fusion Result

$BD_{1}\left(x,y\right)$ and $BD_{2}\left(x,y\right)$ contain most of the energy information from the original image. To avoid excessive energy loss in the fused image, we used the fusion rule of taking the Abs to obtain the background-detail layer of the fused image $BD_{F}\left(x,y\right)$:

(17) $$BD_{F}\left(x,y\right)=\left\{\begin{array}{ll} BD_{1}\left(x,y\right), & BD_{1}\left(x,y\right)\geq BD_{2}\left(x,y\right)\\ BD_{2}\left(x,y\right), & else \end{array}\right..$$

Because the salient-detail layers contain significant information, they are fused by direct summation, as follows:

(18) $$SD_{F}\left(x,y\right)=SD_{1}\left(x,y\right)+SD_{2}\left(x,y\right).$$

where $SD_{F}\left(x,y\right)$ denotes the salient-detail layers of the fused image. Finally, the fused image is obtained by combining the base, salient-detail, and background-detail layers, as follows:

(19) $$I_{F}\left(x,y\right)=B_{F}\left(x,y\right)+BD_{F}\left(x,y\right)+SD_{F}\left(x,y\right).$$

The formal mechanism of the proposed method is described in Algorithm 1.
**Algorithm 1** Steps in proposed fusion method*Inputs*: Medical CT Image $I_{1}$; Medical MRI Image $I_{2}$ *Output*: Fused image F Step 1: The BLF-LS is employed to decompose $I_{1}$ and $I_{2}$ to obtain the corresponding base layers $B_{1}$ and $B_{2}$ and detail layers $D_{1}$ and $D_{2}$ (Equations (6) and (7)). Step 2: The DSC-RGF algorithm is utilized to decompose the detail layers $D_{1}\;\mathrm{and}\;D_{2}$ to obtain the corresponding significant-detail layers $SD_{1}$ and $SD_{2}$ and background-detail layers $BD_{1}$ and $BD_{2}\;$(Equations (8)–(12)). Step 3: The fusion base layer $B_{F}$ is obtained using the MLEN rule (Equations (13)–(16)). Then, the fusion Abs rule is employed to fuse $BD_{1}$ and $BD_{2}$ and thereby obtain the fusion base layer $BD_{F}$ (Equation (17)). $SD_{1}$ and $SD_{2}$ are added to obtain the significant-detail layer $SD_{F}$ of the fused image (Equation (18)). Step 4: The fused image is obtained by summing $GD_{F}$, $B_{F}$, and $SD_{F}$ (Equation (19)).

## 4. Experimental Results and Comparisons

### 4.1. Experimental Setup

#### 4.1.1. Test Data

The experimental dataset was selected from 100 sets each comprising a CT-MRI, PET-MRI, and SPECT-MRI image, amounting to 300 source images for testing. The source images were images of the human brain captured by different imaging mechanisms; each image was 256 × 256 pixels, and each pair of images was aligned. The test images were obtained from a database of Harvard Medical School (http://www.med.harvard.edu/aanlib/home.html, accessed on 8 November 2022).

#### 4.1.2. Quantitative Evaluation Metrics

Subjective quality assessment of image fusion represents human intuition but lacks quantitative description, so objective quality assessment is also needed to evaluate the performance of fusion algorithms. The metrics used for objective quality evaluation of images typically include three categories: information theory-based, image feature-based, and human perception-inspired fusion metrics. In this study, six common metrics were selected to objectively assess the fusion performance: normalized mutual information $\left(Q_{MI}\right)$ [30], image fusion metric based on a multiscale scheme $\left(Q_{M}\right)$ [31], nonlinear correlation information entropy $\left(Q_{NCIE}\right)$ [32], metric based on phase congruency $\left(Q_{P}\right)$ [33], entropy $\left(EN\right)$ [34], and visual information fidelity $\left(VIF\right)$ [35].

$Q_{MI}$ is a quality index that describes the quantity of information conveyed from the source image to the fused image; $Q_{NCIE}$ is used to display the nonlinear correlation degree of the concerned multivariable dataset; EN measures the amount of information contained in the fused image; $Q_{MI}$, $Q_{NCIE}$, and EN are evaluation metrics based on information theory. $Q_{M}$ evaluates the retention value of the edge information in fused images from multiple scales; $Q_{P}$ is defined by the maximum and minimum moments of phase coherence and is used to evaluate the angle and edge information measures; $Q_{M}$ and $Q_{P}$ are evaluation metrics based on image features. The VIF metric measures the information fidelity of the fused image, and the distortions of the images include additive noise, blur, and global or local changes in contrast. VIF is a fusion measure inspired by human perception. Table 1 shows a summary of these six metrics. A comprehensive and objective evaluation of the fused image quality is achieved by considering these metrics, and the larger the value of all these metrics, the better the quality of the fused image [36].

#### 4.1.3. Methods Compared with Proposed Methods

To verify the effectiveness of the proposed methods, our results were compared with those of nine state-of-the-art fusion algorithms, including two SD methods, namely information of interest in local Laplacian filtering (ILLF) [37] and LRD [8]; two TDB methods, namely non-subsampled shearlet transform–pulse coupled neural network (NSST-PCNN) [38] and NSCT–PCLLE [7]; and five DL methods, namely zero-learning fast (Zero-LF) [39], image fusion framework based on CNN (IFCNN) [18], squeeze-and-decomposition network (SDNet) [40], enhanced medical image fusion network (EMFusion) [41], and unified unsupervised image fusion network (U2Fusion) [42]. For a fair comparison, the parameter settings of all the methods were consistent with the original text. The methods ILLF, LRD, TDB, NSST-PCNN, and NSCT-PCLLE were implemented in MATLAB 2019a, methods Zero-LF, IFCNN, SDNet, EMFusion, and U2Fusion were implemented in PyCharm 2022. All of the fusion methods were implemented on a PC with Intel^®^ Core™; i7-5500U 2.40 GHz-CPU (2394 MHz) and 12-GB RAM.

### 4.2. Parameter Analysis

**Figure 4:**
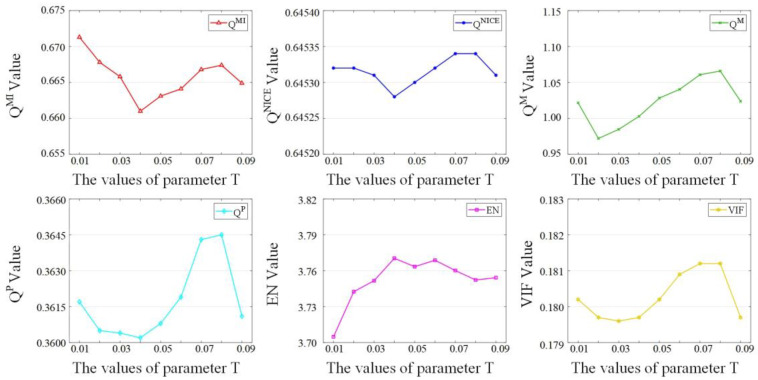
Fusion performance under different values of parameter *T*.

Different parameters determine the performance of the algorithm. In the proposed method, the parameter *T* in Equation (8) plays a decisive role in the performance of the algorithm, mainly because after processing the detail layers using saliency detection to obtain salient information, a decision map (SGF) was needed to extract this information to the salient detail layer. It was found using Equation (11) that there was more salient information when there was more favorable SGF to the salient information layer. According to Equation (8), the threshold value *T* was the key factor affecting the initial salient decision maps, influencing the SGF through Equations (9) and (10). For smaller values of *T*, the SGF was more favorable to the salient detail layers, and more salient information was contained in the salient detail layer. However, when *T* was too small, the noise in the salient detail layer could not be reduced effectively, so we needed a reasonable size of *T*. The selection of the parameter *T* in the proposed model is discussed here. We selected five sets of source images and set the variation range of *T* to 0.01–0.09 data (eight sets in total) because it was difficult to clearly distinguish the differences in quality of these fusion results using only subjective quality assessment. Six metrics were used to evaluate the fusion results, and the average value of the objective evaluation of the five sets of images was obtained, as shown in Figure 4. $Q_{MI}$ has a large value when *T* is 0.01, $Q_{M}$, $Q_{P},$ and VIF have large values when *T* is 0.08, $Q_{NICE}$ has a large value when *T* is 0.07, and EN has a large value when *T* is 0.04. Considering these indicators, we set *T* to 0.07.

For the other parameters, the GRF filter was set to [filter size r = 3, blur degree ε = 0.3, iteration number $T^{\prime }$ = 4]. Based on previous suggestions [27], the BLF-LS was set to [$\sigma_{S}$ = 12, $\sigma_{r}=0.02$], and the window size for the *SML* in Equation (13) and LEN in Equation (15) were set to 3 × 3.

### 4.3. Subjective Quality Assessment

**Figure 5:**
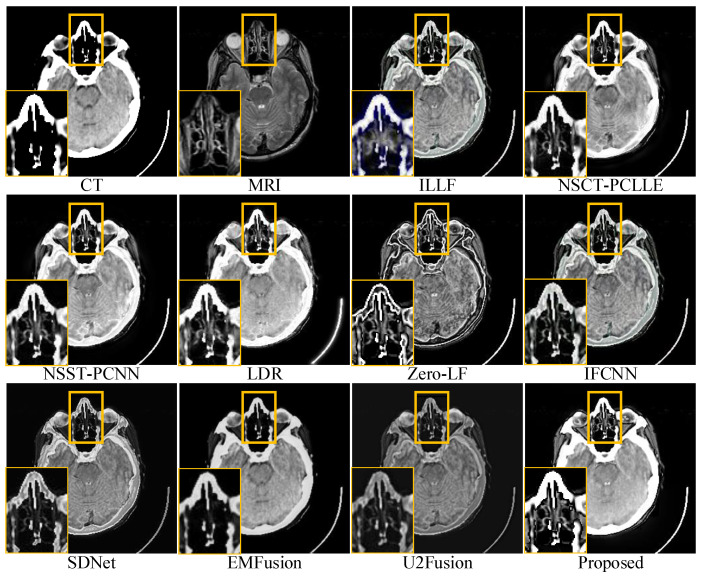
Comparison of performances of various methods on computed tomography magnetic resonance imaging (CT-MRI) source images. For a clear comparison, we select a same region (i.e., the yellow box) in each image and zoom in it in the bottom left corner.

**Figure 6:**
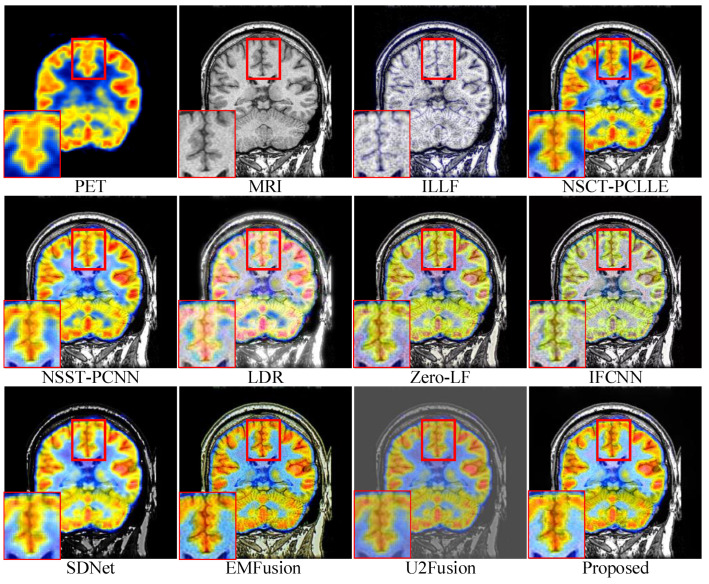
Comparison of performances of various methods on the positron emission tomography (PET)-MRI source images. For a clear comparison, we select a same region (i.e., the red box) in each image and zoom in it in the bottom left corner.

**Figure 7:**
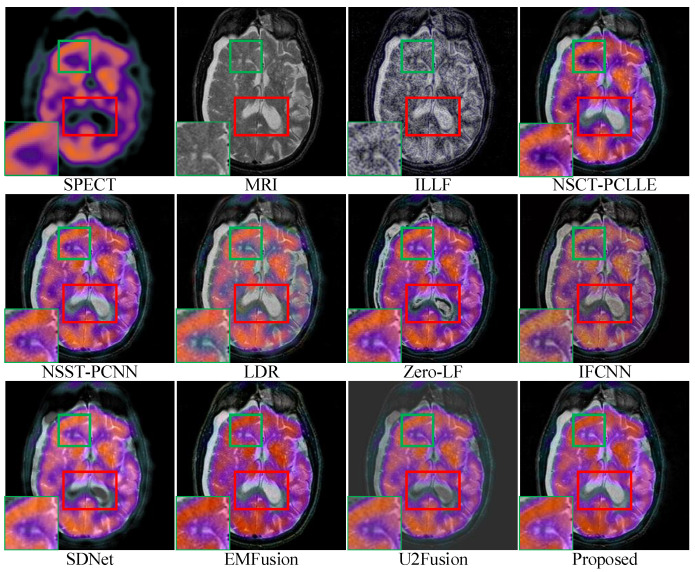
Comparison of performances of various methods on the single-photon emission computed tomography (SPECT)-MRI images. For a clear comparison, we select two same regions (i.e., the green and red boxes) in each image, and the green boxes are zoomed in the bottom left corner.

For conciseness, we have only shown the results of three sets of images in the subjective evaluation. Figure 5, Figure 6 and Figure 7 show the fusion results of different types of medical images obtained by different image fusion algorithms.

The fusion results of the different methods on CT-MRI medical images are shown in Figure 5. The local areas are marked by colored rectangles, which are enlarged in the lower left corners for better comparison. All the methods retained the main information and features, as shown in Figure 5; however, there were still significant differences regarding the features. ILLF showed color distortion, which led to the introduction of speckles in the fusion results. Zero-LF, IFCNN, SDNet, EMFusion, and U2Fusion could not completely retain the energy in the CT images, leading to low brightness and contrast in the fusion results. Second, NSCT-PCLLE, NSST-PCNN, LDR, SDNet, EMFusion, and U2Fusion were unable to retain the detail information in the MRI images (yellow part of the magnification area). Figure 5 shows that the proposed method outperformed the other methods in terms of the energy retention of CT-MRI medical-source images. It also successfully preserved information such as the details and structures in the source images without artifacts and brightness distortion.

Figure 6 shows a set of PET-MRI images fused by different methods. The fusion results of ILLF, LDR, Zero-LF, IFCNN, and U2Fusion show their insufficient ability in retaining the color in the PET images, which led to color distortion in the fusion results. LDR, SDNet, EMFusin, and U2Fusion performed poorly in retaining the luminance information of the MIR images; the luminance-oversaturation phenomenon occurred in the fusion results under LDR. Under SDNet, EMFusion, and U2Fusion, most of the energy from the MRI images was lost, particularly under SDNet, which led to a low overall illuminance in the images. The enlarged portion in the lower left corners shows that our method was able to retain the detailed part of the MIR image. Additionally, under our method, jagged edges can be observed in the fused image, while these edges were slightly missing under the other methods, demonstrating the superior performance of our method. Overall, Figure 6 indicates that our method could retain the structural information of the source image and outperformed the other methods in expressing intensity-based features.

In Figure 7, under ILLF, LDR, IFCNN, and EMFusion, there are deviations in color in the source SPECT image. Regarding the source image, the ILLF results show grayscale information, the LDR and IFCNN results show lighter colors, and the EMFusion results show color enhancement. NSCT-PCLLE, NSST-PCNN, Zero-LF, SDNet, and U2Fusion did not completely capture the luminance information of the MRI images, and this is represented by a small black shading in the marked red area. The fusion results of NSCT-PCLLE, NSST-PCNN, Zero-LF, SDNet, and U2Fusion show black blocks. As shown by the green enlarged area in the lower left corner of the images, ILLF, NSST-PCLLE, LDR, IFCNN, SDNet, and U2Fusion were not completely capable of retaining the details in the source image, while our method was able to retain them well. Figure 7 shows that the images fused under our proposed method are more informative, clearer, and have a higher contrast than those under the existing methods.

### 4.4. Objective Quality Assessment

Table 2, Table 3 and Table 4 show the objective evaluations of the different methods. Table 2 shows the objective evaluation results of the CT-MRI images. Our proposed method ranked first for the indicators $Q_{MI}$, $Q_{NCIE}$, $Q_{M}$, $Q_{P}$ and VIF. This shows that our method obtained good results regarding the amount of information it could transfer from the source image to the fused image, the degree of nonlinear correlation, edge information, phase consistency, and information fidelity. Although it did not rank high for EN, the difference between its value and the highest value was small; therefore, we concluded that the proposed method was able to produce good results for the CT-MRI images under an objective evaluation assessment.

Table 3 and Table 4 show the objective evaluation results of the nine methods on the PET-MRI and MRI-SPECT color images, respectively. Our method did not rank first in certain metrics, but its overall ranking was at the top. It also achieved good results for the PET-MRI and MRI-SPECT images under the objective evaluation assessment.

Based on the above subjective visual evaluation and objective metrics analysis, we concluded that the fusion performance of our method was the highest of all methods. This was mainly due to the good decomposition of the details and basis values of the images from using the BLF-LS, the effective preservation of the significant structure and edge information of the source image in the fused image using saliency detection, and the processing of the weight map using the RGF, which makes full use of the strong correlation between the neighboring pixels.

### 4.5. Discussion on Time Efficiency

In this section, we compared the time efficiency of the proposed method with those of the other nine methods on grayscale images. As shown by the results in Table 5, the Zero-LF, IFCNN, SDNet, EMFusion, and U2Fusion DL methods trained the models in advance, allowing them to process the images quickly. The ILLF method had the longest running time because the ILLF filter was not as fast as the other multi-scale tools and computed the decomposition of the image at different scales. The LRD algorithm took too long in the gradient-domain image enhancement owing to its over-reliance on the fitting function. NSST-PCNN also required more time than our method because of the PCNN iterations that were involved. Although the proposed method was not the fastest, considering its high performance, it was still effective. Moreover, we believe that if we fully optimize the code behind the working of our method and convert it to increase its efficiency using tools such as the graphical processing unit (GPU) and C++, the time required to execute our method will be significantly shorter, enabling the method to satisfy the requirements of more applications.

### 4.6. Extension to Infrared (IR) and Visible (VIS) Image Fusion

**Figure 8:**
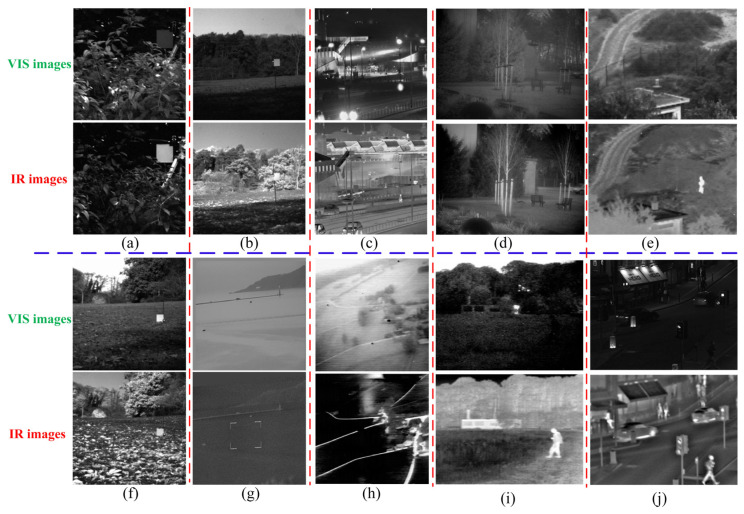
Source images. (**a**–**j**) are the ten pairs of VIS/IF images.

To justify the ability of our proposed method to generalize, we tested the fusion ability of our method on ten sets of IR-VIS images (shown in Figure 8). Six advanced fusion methods for IR-VIS images were selected for comparison: visual saliency map and weighted least square optimization (VSWL) [43], Gaussian curvature filtering (GCF) [44], IFCNN [18], SDNet [40], U2Fusion [42], and SwinFusion [45].

**Figure 9:**
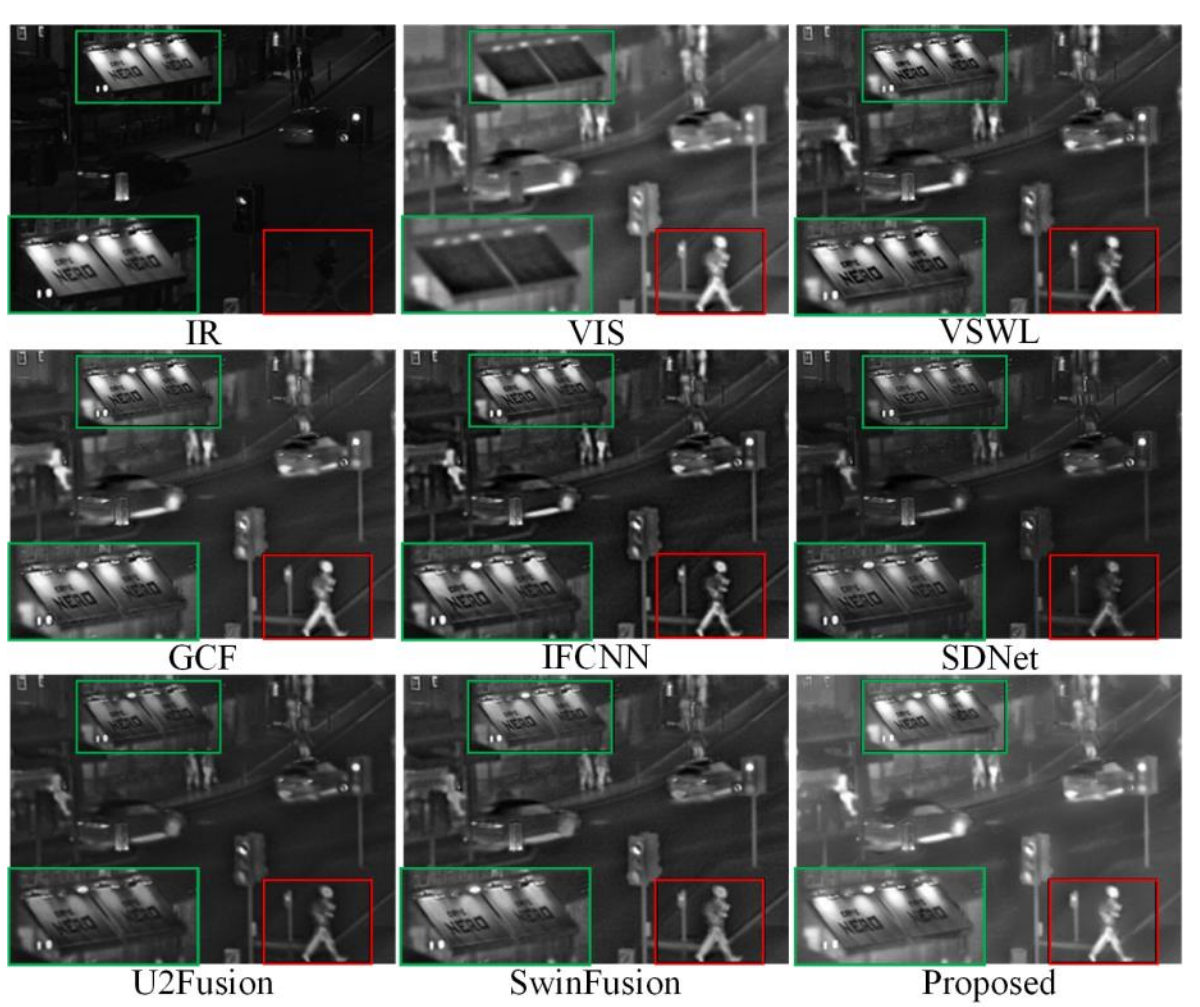
Comparison of performance of various methods on source images of Queens Road, Bristol. For a clear comparison, we select two same regions (i.e., the green and red boxes) in each image, and the green boxes are zoomed in the bottom left corner.

As shown in Figure 9, although all seven methods could retain the energy in the IR image and the details in the VIS image, differences still existed. The red box with the pedestrian in the lower right corner of Figure 9 shows that although all seven methods obtained the detailed and contour information of the person in the source image, the overall brightness, specifically under the SDNet, U2Fusion, and SwinFusion methods, was low. In the fusion results of the proposed methods, the person’s edges did not appear as black shadows owing to the smoothing of the edges of the significant target using RGF. Second, regarding the poster board framed in green in the lower-left corner of the image in Figure 9, the VSWL, GCF, IFCNN, SDNet, U2Fusion, and SwinFusion methods compared the light map without retaining the overall luminance information of the light sign. The above analysis proves that our algorithm had the best detail retention and color fidelity and was more consistent with the subjective vision for processing object edges in an image.

**Figure 10:**
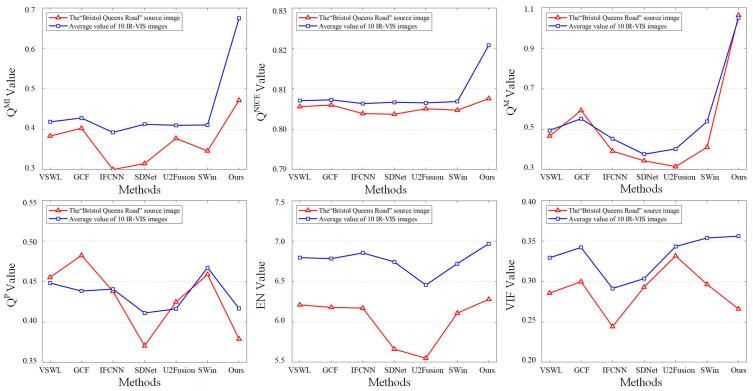
Objective evaluation of 7 methods on IR-VIS image fusion.

Figure 10 shows the objective evaluation results for the “Queens Road, Bristol” image, and the average value of the objective evaluation of the 10 IR-VIS images (the 10 sets of images shown in Figure 8). The horizontal and vertical coordinates represent the different methods and the values of different evaluation metrics, respectively. The red line shows the objective evaluation of the different methods on the image “Queens Road, Bristol”, and the blue line shows the average value of the objective evaluation of the different methods in Figure 8. Regarding the objective evaluation assessment, our method ranked first in $Q_{MI}$, $Q_{NCIE}$, $Q_{M}$, EN, and the average VIF index for the 10 IR-VIS images. Although the result for $Q_{P}$ was not the highest, its difference from the best value was not pronounced. Thus, the validity of the proposed method in terms of objective assessment is confirmed. The above evaluation shows that our method can be effectively extended to IR-VIS image fusion.

## 5. Conclusions

In this study, we proposed a multi-sensor medical-image fusion method based on the BLF-LS and DSC. First, the image decomposition of the base layer using the BLF-LS was simple and effective as it potentially captured large changes in intensity. The detail layer preserved details such as the structure, texture, and edges of the original image efficiently. The DCS model effectively detected the salient information, and the GRF made full use of the strong correlation between the neighboring pixels for weight optimization, allowing the fused detail layer to effectively retain the salient structure and edge information in the source image. Finally, the base-layer fusion rules based on the MLEN effectively preserved the energy information of the source images.

The fusion results of different methods on CT-MRI, PET-MRI and MRI-SPECT images were demonstrated. The experimental results showed the advantages of the proposed method in both subjective visual and objective quantitative evaluations. Compared to the nine state-of-the-art methods used in the study, the proposed medical image fusion algorithm can provide fusion images with clearer edge details, complete salient information, more brightness, and superior colors. Additionally, this method is also applicable to IR-VIS image fusion. However, the proposed fusion method is easily affected by noise because the inputs are in alignment pairs. In the future, we will work on solving the effect of noise on images, thus bridging the gap between medical image fusion and actual clinical applications.

## Figures and Tables

**Table 1:** Quantitative evaluation metrics used in the experiments.

	Metric	Mathematical Expression	Definition	Best Value Outcomes
1	$Q_{MI}$ [30]	$Q_{MI}=2\left[\frac{MI\left(A,F\right)}{H\left(A\right)+H\left(F\right)}+\frac{MI\left(B,F\right)}{H\left(B\right)+H\left(F\right)}\right]$	Measure of retention value of edge information	Higher
2	$Q_{NCIE}$ [32]	$Q_{NCIE}=1+\sum_{i=1}^{3}\frac{\lambda_{i}}{3}{log}_{b}\frac{\lambda_{i}}{3}$	Measure of nonlinear correlation information entropy	Higher
3	$Q_{M}$ [31]	$Q_{M}=\prod_{s=1}^{N}{\left(Q_{s}^{\frac{AB}{F}}\right)}^{\alpha_{s}}$	Measure of retention value of edge information	Higher
4	$Q_{P}$ [33]	$Q_{P}={\left(P_{p}\right)}^{\alpha }{\left(P_{M}\right)}^{\beta }{\left(P_{m}\right)}^{\gamma },$	Measure of phase congruency	Higher
5	EN [34]	$EN=-\sum_{l=0}^{L-1}p_{i}{log}_{2}p_{i}$	Evaluation metrics based on information theory	Higher
6	VIF [35]	$VIF=\frac{\sum_{j\in subbands}I({\vec{C}}^{N,j};{\vec{F}}^{N,j}|s^{N,j})}{\sum_{j\in subbands}I({\vec{C}}^{N,j};{\vec{E}}^{N,j}|s^{N,j})}$	A fusion measure inspired by human perception	Higher

**Table 2:** Comparison of performance of proposed fusion method with those of nine existing methods on CT-MRI images; the best results are shown in bold.

	Methods	$Q_{MI}$	$Q_{NCIE}$	$Q_{M}$	$Q_{P}$	EN	VIF
**Objective evaluation of different fused images in Figure 5**	ILLF	0.5554	0.8054	0.1583	0.2049	5.7517	0.1330
	NSST-PCNN	0.6502	0.8050	0.6791	0.3444(3)	4.7473	0.2048
	NSCT-PCLLE	0.6232	0.8047	0.6962(3)	0.2792	4.6168	0.2194(3)
	LRD	0.7302	0.8056(3)	0.2218	0.3292	4.5160	0.1702
	Zero-LF	0.7918(3)	0.8056	**1.5481**	0.1172	4.0112	0.0874
	IFCNN	0.6690	0.8047	0.1469	0.2907	4.2017	0.1669
	SDNet	0.6513	0.8051	0.1084	0.2840	4.8154(2)	0.1712
	EMFusion	0.8345(2)	0.8063(2)	0.1309	**0.5829**	4.2328	**0.3247**
	U2Fusion	0.6059	0.8045	0.0849	0.2925	4.4836	0.1862
	Proposed	**0.8443**	**0.8070**	1.1038(2)	0.4424(2)	4.7689(3)	0.2293(2)
**Average evaluation mean of 100 groups of images**	ILLF	0.7265	0.8048	0.1635	0.2890	**4.1261**	0.2087
	NSST-PCNN	0.7532	0.8049	0.6157	0.2896	3.9475	0.2488
	NSCT-PCLLE	0.7319	0.8048	0.7197(3)	0.2726	3.9428	0.2736(3)
	LRD	0.7861	0.8052	0.3345	0.2548	3.8813	0.1994
	Zero-LF	0.8749(3)	0.8057(3)	**1.6937**	0.1465	3.6424	0.0993
	IFCNN	0.7512	0.8047	0.1797	0.2904	3.7007	0.1915
	SDNet	0.7578	0.8052	0.1282	0.2713	4.1732(2)	0.1928
	EMFusion	0.8933(2)	0.8057(2)	0.1510	0.4295	3.6298	0.3504
	U2Fusion	0.6976	0.8045	0.1138	0.3130	3.9285	0.2249
	Proposed	**0.8935**	**0.8064**	1.1555 (2)	0.3820 (2)	4.0829(3)	0.2903(2)

**Table 3:** Comparison of performance of proposed fusion method with those of nine methods on PET-MRI images; the best results are shown in bold.

	Methods	$Q_{MI}$	$Q_{NCIE}$	$Q_{M}$	$Q_{P}$	EN	VIF
**Objective evaluation of performance for images in Figure 6**	ILLF	0.3732	0.8035	0.0547	0.0167	4.6800	0.0095
	NSST-PCNN	0.6509(2)	0.8078(2)	1.4767(2)	0.4576	5.8536	0.2977
	NSCT-PCLLE	0.6040	0.8069	1.3982(3)	0.4276	5.9460(3)	0.2982(3)
	LRD	0.5225	0.8060	0.2486	0.3397	**6.4027**	0.1808
	Zero-LF	0.6229(3)	0.8074(3)	0.3195	0.4543	5.6813	0.2352
	IFCNN	0.6073	0.8072	0.2155	0.4949(2)	5.8007	0.2461
	SDNet	0.6062	0.8057	0.1035	0.2804	5.0403	0.1451
	EMFusion	0.5906	0.8072	0.2717	**0.5669**	5.8329	0.2688
	U2Fusion	0.5653	0.8055	0.0879	0.3689	5.0805	0.3071(2)
	Proposed	**0.8319**	**0.8129**	**1.9020**	0.4902(3)	6.2604(2)	**0.3170**
**Average evaluation mean of 100 groups of images**	ILLF	0.3448	0.8035	0.1230	0.0956	5.1814	0.0503
	NSST-PCNN	0.7019(2)	0.8088(3)	1.6547(2)	0.5141	5.7223	0.3330
	NSCT-PCLLE	0.6635(3)	0.8081	1.6272(3)	0.4939	5.7743	**0.3369**
	LRD	0.5533	0.8064	0.3332	0.3583	**6.2651**	0.1920
	Zero-LF	0.6558	0.8077	0.6523	0.4809	5.6436	0.2434
	IFCNN	0.6187	0.8072	0.3018	0.5403(2)	5.6759	0.2572
	SDNet	0.6235	0.8057	0.1288	0.2815	4.9340	0.1528
	EMFusion	0.6160	**0.8242**	0.3305	**0.6479**	5.8601(3)	0.2819
	U2Fusion	0.5753	0.8055	0.1161	0.4202	4.9477	0.3341(3)
	Proposed	**0.8836**	0.8134(2)	**2.0628**	0.5148(3)	5.9378(2)	0.3342(2)

**Table 4:** Comparison of performance of proposed fusion method with those of nine methods on MRI-SPECT images; the best results are shown in bold.

	Methods	$Q_{MI}$	$Q_{NCIE}$	$Q_{M}$	$Q_{P}$	EN	VIF
**Objective evaluation of different fused images in Figure 7**	ILLF	0.5339	0.8063	0.2813	0.2664	5.8953	0.1818
	NSST-PCNN	0.6030	0.8073	1.0805(3)	0.3283	6.0048(2)	0.3171
	NSCT-PCLLE	0.5940	0.8072	1.0262	0.3452(3)	5.9899(3)	0.3561(2)
	LRD	0.6423	0.8081	0.7759	0.3395	**6.1174**	0.3051
	Zero-LF	0.6972(3)	0.8087(3)	1.3326(2)	0.3233	5.7236	0.2191
	IFCNN	0.5995	0.8069	0.5536	0.3206	5.6857	0.2846
	SDNet	0.6525	0.8074	0.2431	0.2395	5.6691	0.2214
	EMFusion	0.7402(2)	0.8102(2)	0.6409	**0.5500**	5.6414	**0.3927**
	U2Fusion	0.5883	0.8063	0.2151	0.2791	5.2268	0.3342(3)
	Proposed	**0.8526**	**0.8132**	**1.4879**	0.4283(2)	5.9386(4)	0.3226(4)
**Average evaluation mean of 100 groups of images**	ILLF	0.5761	0.7898	0.2838	0.3237	5.1492	0.1933
	NSST-PCNN	0.6856	0.7914	1.1394(3)	0.4271	5.1601(3)	0.3365
	NSCT-PCLLE	0.6908	0.7915	1.0820	0.4422(3)	5.1117	0.3657(3)
	LRD	0.6687	0.7912	0.6880	0.4002	**5.3850**	0.2965
	Zero-LF	0.7677(2)	0.7921(3)	1.3337(2)	0.3971	4.8929	0.2486
	IFCNN	0.6542	0.7903	0.5443	0.4083	4.8430	0.2917
	SDNet	0.7127	0.7904	0.2070	0.2964	4.5649	0.2427
	EMFusion	0.7638(3)	0.7927(2)	0.5768	**0.6211**	4.8813	**0.3799**
	U2Fusion	0.6307	0.7896	0.1951	0.3483	4.4321	0.3703(2)
	Proposed	**0.8736**	**0.7955**	**1.4704**	0.4701(2)	5.2540(2)	0.3194(5)

**Table 5:** Running times of different methods for fusing two source images of 256 × 256 pixels each.

Methods	ILLF	NSST-PCNN	NSCT-PCLLE	LRD	Zero-LF
Time	161.51	10.01	3.26	126.64	2.45
**Methods**	**IFCNN**	**SDNet**	**EMFusion**	**U2Fusion**	**Proposed**
Time	0.21	0.16	0.57	0.36	4.24

## Data Availability

The data presented in this study are available upon request from the authors.

## References

[1] Goyal B., Dogra A., Khoond R., Gupta A., Anand R. Infrared and Visible Image Fusion for Concealed Weapon Detection using Transform and Spatial Domain Filters. Proceedings of the 2021 9th International Conference on Reliability, Infocom Technologies and Optimization (Trends and Future Directions) (ICRITO).

[2] Hermessi H., Mourali O., Zagrouba E. (2021). Multimodal medical image fusion review: Theoretical background and recent advances. Signal Process..

[3] Li S.T., Kang X.D., Fang L.Y., Hu J.W., Yin H.T. (2017). Pixel-level image fusion: A survey of the state of the art. Inf. Fusion.

[4] Zhang Q., Liu Y., Blum R.S., Han J.G., Tao D.C. (2018). Sparse representation based multi-sensor image fusion for multi-focus and multi-modality images: A review. Inf. Fusion.

[5] Zhang H., Xu H., Tian X., Jiang J.J., Ma J.Y. (2021). Image fusion meets deep learning: A survey and perspective. Inf. Fusion.

[6] Li X.S., Zhou F.Q., Tan H.S., Chen Y.Z., Zuo W.X. (2021). Multi-focus image fusion based on nonsubsampled contourlet transform and residual removal. Signal Process..

[7] Zhu Z.Q., Zheng M.G., Qi G.Q., Wang D., Xiang Y. (2019). A Phase Congruency and Local Laplacian Energy Based Multi-Modality Medical Image Fusion Method in NSCT Domain. IEEE Access.

[8] Li X., Guo X., Han P., Wang X., Li H., Luo T. (2020). Laplacian Redecomposition for Multimodal Medical Image Fusion. IEEE Trans. Instrum. Meas..

[9] Khan H., Sharif M., Bibi N., Usman M., Haider S.A., Zainab S., Shah J.H., Bashir Y., Muhammad N. (2020). Localization of radiance transformation for image dehazing in wavelet domain. Neurocomputing.

[10] Juneja S., Anand R. (2018). Contrast Enhancement of an Image by DWT-SVD and DCT-SVD.

[11] Li X.S., Zhou F.Q., Tan H.S. (2021). Joint image fusion and denoising via three-layer decomposition and sparse representation. Knowl.-Based Syst..

[12] Li S.T., Yin H.T., Fang L.Y. (2012). Group-Sparse Representation With Dictionary Learning for Medical Image Denoising and Fusion. IEEE Trans. Biomed. Eng..

[13] Zhang Q., Levine M.D. (2016). Robust Multi-Focus Image Fusion Using Multi-Task Sparse Representation and Spatial Context. IEEE Trans. Image Process..

[14] Wang J., Peng J.Y., Feng X.Y., He G.Q., Fan J.P. (2014). Fusion method for infrared and visible images by using non-negative sparse representation. Infrared Phys. Technol..

[15] Gu S., Meng D., Zuo W., Zhang L. Joint Convolutional Analysis and Synthesis Sparse Representation for Single Image Layer Separation. Proceedings of the 2017 IEEE International Conference on Computer Vision (ICCV).

[16] Jie Y., Zhou F., Tan H., Wang G., Cheng X., Li X. (2022). Tri-modal medical image fusion based on adaptive energy choosing scheme and sparse representation. Measurement.

[17] Muhammad N., Bibi N., Jahangir A., Mahmood Z. (2018). Image denoising with norm weighted fusion estimators. Pattern Anal. Appl..

[18] Zhang Y., Liu Y., Sun P., Yan H., Zhao X.L., Zhang L. (2020). IFCNN: A general image fusion framework based on convolutional neural network. Inf. Fusion.

[19] Ma J.Y., Yu W., Liang P.W., Li C., Jiang J.J. (2019). FusionGAN: A generative adversarial network for infrared and visible image fusion. Inf. Fusion.

[20] Luo X., Gao Y., Wang A., Zhang Z., Wu X.J. (2021). IFSepR: A general framework for image fusion based on separate representation learning. IEEE Trans. Multimed..

[21] Zhu Z.Q., He X.Y., Qi G.Q., Li Y.Y., Cong B.S., Liu Y. (2023). Brain tumor segmentation based on the fusion of deep semantics and edge information in multimodal MRI. Inf. Fusion.

[22] Liu Y., Chen X., Wang Z.F., Wang Z.J., Ward R.K., Wang X.S. (2018). Deep learning for pixel-level image fusion: Recent advances and future prospects. Inf. Fusion.

[23] Mo Y., Kang X.D., Duan P.H., Sun B., Li S.T. (2021). Attribute filter based infrared and visible image fusion. Inf. Fusion.

[24] Wang G.F., Li W.S., Du J., Xiao B., Gao X.B. (2022). Medical Image Fusion and Denoising Algorithm Based on a Decomposition Model of Hybrid Variation-Sparse Representation. IEEE J. Biomed. Health Inform..

[25] Xu G.X., Deng X.X., Zhou X.K., Pedersen M., Cimmino L., Wang H. (2022). FCFusion: Fractal Componentwise Modeling With Group Sparsity for Medical Image Fusion. IEEE Trans. Ind. Inform..

[26] Li X.S., Zhou F.Q., Tan H.S., Zhang W.N., Zhao C.Y. (2021). Multimodal medical image fusion based on joint bilateral filter and local gradient energy. Inf. Sci..

[27] Liu W., Zhang P.P., Chen X.G., Shen C.H., Huang X.L., Yang J. (2020). Embedding Bilateral Filter in Least Squares for Efficient Edge-Preserving Image Smoothing. IEEE Trans. Circuits Syst. Video Technol..

[28] Wu X., Ma X., Zhang J., Wang A., Jin Z. Salient Object Detection Via Deformed Smoothness Constraint. Proceedings of the 2018 25th IEEE International Conference on Image Processing (ICIP).

[29] Huang W., Jing Z. (2007). Evaluation of focus measures in multi-focus image fusion. Pattern Recognit. Lett..

[30] Qu G.H., Zhang D.L., Yan P.F. (2002). Information measure for performance of image fusion. Electron. Lett..

[31] Wang P.W., Liu B. A Novel Image Fusion Metric Based on Multi-Scale Analysis. Proceedings of the 9th International Conference on Signal Processing.

[32] Wang Q., Shen Y., Zhang J.Q. (2005). A nonlinear correlation measure for multivariable data set. Phys. D-Nonlinear Phenom..

[33] Zhao J.Y., Laganiere R., Liu Z. (2007). Performance assessment of combinative pixel-level image fusion based on an absolute feature measurement. Int. J. Innov. Comput. Inf. Control.

[34] Ma J.Y., Ma Y., Li C. (2019). Infrared and visible image fusion methods and applications: A survey. Inf. Fusion.

[35] Sheikh H.R., Bovik A.C. (2006). Image information and visual quality. IEEE Trans. Image Process..

[36] Liu Z., Blasch E., Xue Z.Y., Zhao J.Y., Laganiere R., Wu W. (2012). Objective Assessment of Multiresolution Image Fusion Algorithms for Context Enhancement in Night Vision: A Comparative Study. IEEE Trans. Pattern Anal. Mach. Intell..

[37] Du J., Li W.S., Xiao B. (2017). Anatomical-Functional Image Fusion by Information of Interest in Local Laplacian Filtering Domain. IEEE Trans. Image Process..

[38] Yin M., Liu X.N., Liu Y., Chen X. (2019). Medical Image Fusion With Parameter-Adaptive Pulse Coupled Neural Network in Nonsubsampled Shearlet Transform Domain. IEEE Trans. Instrum. Meas..

[39] Lahoud F., Süsstrunk S. Zero-Learning Fast Medical Image Fusion. Proceedings of the 2019 22th International Conference on Information Fusion (FUSION).

[40] Zhang H., Ma J.Y. (2021). SDNet: A Versatile Squeeze-and-Decomposition Network for Real-Time Image Fusion. Int. J. Comput. Vis..

[41] Xu H., Ma J.Y. (2021). EMFusion: An unsupervised enhanced medical image fusion network. Inf. Fusion.

[42] Xu H., Ma J.Y., Jiang J.J., Guo X.J., Ling H.B. (2022). U2Fusion: A Unified Unsupervised Image Fusion Network. IEEE Trans. Pattern Anal. Mach. Intell..

[43] Ma J.L., Zhou Z.Q., Wang B., Zong H. (2017). Infrared and visible image fusion based on visual saliency map and weighted least square optimization. Infrared Phys. Technol..

[44] Tan W., Zhou H.X., Song J.L.Q., Li H., Yu Y., Du J. (2019). Infrared and visible image perceptive fusion through multi-level Gaussian curvature filtering image decomposition. Appl. Opt..

[45] Ma J.Y., Tang L.F., Fan F., Huang J., Mei X.G., Ma Y. (2022). SwinFusion: Cross-domain Long-range Learning for General Image Fusion via Swin Transformer. IEEE-CAA J. Autom. Sin..

